# Understanding suicidal ideation–a network analysis of the Interpersonal Needs Questionnaire

**DOI:** 10.1371/journal.pone.0293026

**Published:** 2023-11-13

**Authors:** Katharina Conrad, Thomas Forkmann, Dajana Schreiber, Tobias Teismann, Heide Glaesmer, Lena Spangenberg, Antje Schönfelder, Nina Hallensleben, Laura Paashaus, Georg Juckel, Inken Höller

**Affiliations:** 1 Department of Clinical Psychology, University of Duisburg-Essen, Essen, Germany; 2 Faculty of Psychology, Mental Health Research and Treatment Center, Ruhr-University of Bochum, Bochum, Germany; 3 Department of Medical Psychology and Medical Sociology, University Leipzig, Leipzig, Germany; 4 Department of Psychiatry, LWL-University Hospital, Ruhr-University of Bochum, Bochum, Germany; 5 Department of Clinical Psychology and Psychotherapy, Charlotte Fresenius Hochschule, Düsseldorf, Germany; Universidad de Zaragoza, SPAIN

## Abstract

**Introduction:**

Identifying various interacting risk factors for suicidality is important to develop preventive measures. The *Interpersonal-Psychological Theory of Suicidal Behavior (IPTS)* postulates suicidal ideation resulting from the occurrence of *Perceived Burdensomeness (PB)* and *Thwarted Belongingness (TB)*. Suicidal behavior ultimately occurs if people have a *Capability for Suicide*. In past studies, the validity of TB was often not empirically confirmed, questioning which of the aspects of TB are central and related to suicidal ideation and whether applied measurement methods adequately capture the construct.

**Method:**

Using a sample of 3,404 individuals from different clinical and nonclinical settings, 30% (1,023) of whom reported suicidal ideation, two network analyses were conducted on the *Interpersonal Needs Questionnaire (INQ)* and a variable mapping suicidal ideation.

**Results:**

Analyses revealed that some items of the INQ were not related to suicidal ideation and the most central items did not have the strongest associations to suicidal ideation.

**Conclusion:**

Based on these results, a shortened version of the INQ with the four items that showed the strongest associations with suicidal ideation in the network analyses was suggested.

## Introduction

More than 700,000 people die by suicide each year worldwide [1]. The lifetime prevalence of suicidal ideation was reported to be as high as 33% among adults [2]. Thus, suicidal ideation as well as suicidal behavior are significant problems for the global health care system [3]. For this reason, both a comprehensive understanding of suicide and the identification of protective and risk factors are important to develop preventive methods and interventions [4].

Both suicidal ideation and suicidal behavior are complex phenomena with various interacting personal, social, psychological, cultural, biological, and environmental factors [5] that determine the individual suicide risk as the result of a rather complex interplay of different risk- and protective factors [3]. Mental disorders, particularly depressive disorders, have been shown to be among the strongest risk factors for suicide attempts and suicide deaths [4]. One of the most influential theories in this regard is the *Interpersonal-Psychological Theory of Suicidal Behavior* [IPTS; 6,7]. The IPTS postulates that suicidal ideation results from the occurrence of two components, *Perceived Burdensomeness* (feeling like a burden to others; PB) and *Thwarted Belongingness* (feeling as if one does not belong; TB). PB refers to the misestimation of individuals that their death is more worth to others than their life. TB is derived from the *Need to Belong* [8]. The need can be satisfied by the presence of frequent, positive social interactions. In the context of the IPTS, the failure of satisfying the Need to Belong is described through TB. The passive desire to die eventually arises [9]. Suicidal behavior ultimately results by adding a third component, *Capability for Suicide* (reduced fear of death, increased pain tolerance). Past research has examined various aspects of the theory in diverse populations [3]. Some studies have been able to confirm the hypotheses that TB and PB are predictors of suicidal ideation [e.g., 10–12]. Other studies yielded conflicting findings; in particular, the validity of TB often could not be empirically confirmed [e.g., 3,13]. Aside from that, there is evidence of non-invariance across certain groups in some groups regarding the predictors of the IPTS. Gender-specific differences were found in studies by Pérez Rodríguez et al. [14] and Seo [15]. A study by Freedenthal et al. [16] found higher levels of PB in men. Other studies could not confirm the gender differences [17–19]. In a study by Upegui-Arango et al. [20] there were differences between clinical and non-clinical populations. However, these differences could not be confirmed in other studies [21,22].

Based on the findings described above, PB seems to be a more reliable predictor of suicidal ideation than TB. However, the inconsistent findings for TB could also be due to the measurement instruments used to examine TB. Commonly, the *Interpersonal Needs Questionnaire* [INQ; 11] is used to measure both TB and PB, as it is a frequently evaluated measurement instrument with good psychometric properties [23]. Yet, there is a possibility that the subscale measuring TB in the INQ does not fully represent the construct [3]. This had already been assumed in other research, finding that TB had no effects on suicidal ideation when measured directly (e.g., with the INQ); however, when using a proxy (perceived social support from others), corresponding effects were found [24]. Vice versa, the method for operationalizing SI could also affect the predictive power of PB and TB. Passed have often used the Beck Scale for Suicide Ideation [BSS; 25] or the Suicidal Behaviors Questionnaire–Revised [SBQ-R; 26] to depict SI. Studies using these tools to examine the impact of TB and PB on SI yielded mixed results, as well. A study by Van Orden et al. [21] using the BSS to measure SI showed that the TB significantly predicted SI scores, while PB did not demonstrate a significant relation with SI. In contrast, a network analysis by De Beurs [27], also using the BSS to operationalize SI, showed that PB and TB both correlated with SI. A meta-analysis by Ma et al. [3] equally reveals varying results for studies that used the SBQ-R as a basis for measuring SI: PB and TB were not always shown to be significant predictors of SI.

Thus, it remains unclear which of the items of the INQ–and especially the TB subscale–are related to suicidal ideation and to what extent, and how these components interact. Furthermore, it is unclear whether TB is not a valid predictor of suicidal ideation, or whether it is due to the INQ inadequately operationalizing the construct, which would call for optimizing the instrument.

To answer these questions, network analyses offer a promising approach. Network analyses provide a way to both quantify and visualize the complex interplay between the items of the INQ and suicidal ideation [28]. On the one hand, predictors of suicidal ideation within the network can be identified on an item level [29]. On the other hand, it is also possible to determine the extent to which (again, on item level) the predictors interact, and which items have a direct effect on suicidal ideation after partialling out effects of the other items [30]. For this purpose, correlation matrices including partial correlations to control for spurious correlations are used as a basis. These can then be visualized as a network [27].

The main goal of this study was, therefore, to test two networks. In the first network, the validity of TB as a predictor of suicidal ideation was reviewed on an item level. Further, a second network to examine the centrality of all single items of the INQ and to illustrate the items’ associations with suicidal ideation was constructed.

To compare the items of the INQ in the network with the items that have turned out to be most central in past research, a synopsis was conducted. For this purpose, various published studies [9,16,21,31,32] validating the INQ with factor analyses were aggregated. The studies were conducted between 2009 and 2016 using the original English versions. A study by Hill et al. [33] validating different versions of the INQ had to be excluded from the synopsis since no factor loadings were provided. Subsequently, the merged factor loadings were averaged and sorted in descending order. The three items 8 ("These days, I feel like I belong." λ = 0.806), 10 ("These days, I am fortunate to have many caring and supportive friends." λ = 0.770), and 14 ("These days, I am close to other people." λ = 0.763) showed the highest factor loadings overall. Being the three items with the highest factor loadings in past studies, as a first hypothesis we assume that they should be the most central ones in the subscale TB of the INQ. Second, we also expect associations between the subscales TB and PB as well as associations with suicidal ideation.

## Materials & methods

### Sample

The raw data used for the analysis (*N* = 3,433) were aggregated from a total of five different samples from different projects.

The first sample (*n* = 2,513) was compiled from a German population-based representative survey on behalf of the University of Leipzig from March to May 2015, with respondents aged 14 to 94 [e.g., 34]. The average age was 49.2 years (*SD* = 18.1), the proportion of male participants was 44.7%.

The second data set (*n* = 67) was taken from the AMBAS project (*Temporal dynamics and acute risk factors of suicidal ideation—A real-time analysis with the Experience Sampling Method (ESM) in inpatients with major depression*) at the University of Leipzig [e.g., 35]. For this purpose, patients with unipolar depression and present suicidal ideation were recruited in inpatient psychiatric units of three German hospitals. Data was collected from August 2015 to July 2017. Patients ranged in age from 18 to 85 years, with a mean age of 37.6 years (*SD* = 14.3). The percentage of female participants was 71.6%. A total of 33.8% of participants reported at least one suicide attempt in the past.

The third sample (*n* = *3*08) consisted of acutely suicidal patients hospitalized at the time of the survey. Data of this sample was collected as part of the project PRESS (*Predictors of the Development of Suicidal Thoughts and Behavior in Longitudinal Section*: *An Evaluation of the Interpersonal Theory of Suicidal Behavior in High-Risk Patients*) at the University of Duisburg-Essen, the Ruhr University Bochum, and the University of Leipzig [e.g., 36]. Data was collected in 13 psychiatric wards of German hospitals from September 2016 to March 2019. The age range was between 18 and 81 years. The average age of the patients was 36.9 years (*SD* = 14.3), 53.6% of the participants were female.

The data of the fourth sample (*n* = 429) was collected as part of the BAM project (*Validation of the Brief Agitation Measure*) at the University of Duisburg-Essen [e.g., 37]. Data collection took place between November 2019 and March 2020 via an anonymous online survey. Participants were on average 27.36 years old (*SD* = 9.67), with an age range of 18 to 81 years. There was a total of 82.3% female participants. 47.3% of the respondents had suffered from a mental illness in the past or at the time of the survey.

The fifth data set (*n* = 116) contains data from a survey of former outpatients who started therapy at a university outpatient clinic in the Ruhr area between April and December 2017 [e.g., 38]. The respondents were on average 39.09 years old (*SD* = 14.53), with an age range of 18 to 78 years. 17.6% of the respondents were male, 27.5% percent of the respondents were female. The remaining respondents did not give any information on this.

After merging the data, *N* = 3,404 (mean age of 44.8 years (*SD* = 16.5); 59% female) cases could be used for the statistical analyses.

### Operationalization of the predictors

The German version of the INQ-15 [39,40] was used to operationalize the two predictors PB and TB. The INQ-15 was established as an empirically derived improvement of the original version INQ-25 [11] and shows stable psychometric properties across multiple studies [21,33]. The questionnaire includes six items measuring PB and nine items measuring TB. The two-factorial structure has been empirically tested and confirmed in various studies on clinical as well as non-clinical samples of different age groups [21]. Items are rated on a seven-point Likert scale from 1 (*not true at all for me)* to 7 (*very true for me)*. There are reversed coded items that have been recoded before conducting the analyses. Higher scores indicate higher feelings of PB and TB. The scale provides good internal consistency [α = .81 for the PB subscale, α = .86 for the TB subscale; 16]. The intercorrelation of the two subscales TB and PB lies in the middle range [r = .50; 16]. Thus, they are most likely related but different constructs.

### Operationalization of suicidal ideation

For the network analyses, it was necessary to capture the participants’ suicidal ideation. For this purpose, a dichotomous variable depicting the presence of lifetime suicidal ideation of the respondents was generated (*S* = 0 if there was/is no suicidal ideation, *S* = 1 if there was/is suicidal ideation).

As the present sample was aggregated from subsamples generated in different projects, partly different methods of measuring suicidal ideation were used. The first data set assembled by the University of Leipzig and the AMBAS project used the *Suicide Behaviors Questionnaire-Revised* [SBQ-R; 26] to operationalize suicidal ideation. The first item refers to suicidal ideation or attempts ("Have you ever thought about or attempted to kill yourself?") If it was answered with "Never.", it was coded with *S* = 0; for any other responses it was coded *S* = 1.

In the PRESS project, only suicidal patients were interviewed. For this reason, all respondents from this project were assigned the value 1 in the variable *S*.

In the BAM project and the Ruhr University Bochum surveys the *Suicidal Ideation and Behavior Scale* [SSEV; 38] was used to measure suicidal ideation and behavior. The scale inquires suicidality in the preceding four weeks on a six-point Likert scale from 0 (*never*) to 6 (*several times a day*). In total, the questionnaire contains six items. To operationalize suicidal ideation, respondents who scored at least a summed value of 1 on the scale were assigned a value of 1 in *S*, otherwise they were assigned the value 0.

### Network analyses

The different source data were merged, and the variable operationalizing suicidal ideation was created with *SPSS Statistics 27* [41]. To clean the data, subjects who had answered less than 80% of the questions (*n* = 29) were excluded from the data set. Missing data of the remaining respondents were then replaced by the respective scale mean.

The program *R* [Version 1.4.1103; 42] was used to perform the correlation analyses. Since the items of the INQ-15 as a rating scale are interval-scaled, the function *cor_auto* in R was used. There is a high degree of multicollinearity between the individual items of the INQ-15, given that items of one construct typically are highly interrelated. Therefore, when performing both network analyses, matrices consisting of partial correlations were created. These reflect the correlations between each two nodes after removing the proportions of variance of all other nodes (or items) in the network. For this purpose, the package *qgraph* [43] was used. In this process, small, non-significant values are automatically set equal to 0. Accordingly, the package does not show any p-values in its output.

The visualization of the networks was also done via *qgraph* in R. For this purpose, the *Fruchterman-Reingold algorithm* [44] was used. The algorithm maps nodes with stronger intercorrelations closer to each other than nodes with lower associations.

First, a network consisting of the nine items of the TB scale was generated to reveal the most central items. As a centrality measure for each node, we used the Strength-index, representing the sum of the sizes of all its edges. The two indices of Closeness and Betweenness have been found to be unstable in past research [45–47], so they were not integrated in the analyses.

In a second network, we further integrated all 15 items of the INQ-15 as well as the variable S operationalizing suicidal ideation. Similarly, in this network the most central items of the INQ-15 were mapped through their Strength. Additionally, the items with the strongest correlations to the suicidal variable were determined.

For better comparability, the Strength was z-standardized (*M* = 0, *SD* = 1) ranging from -3 to 3. A positive value means that an item is more central compared to the average of all items, a negative value implies a lower centrality compared to the average centrality of all items.

## Results

Overall, 30% (*n* = 1,023) of respondents of the entire sample reported suicidal ideation, 70% (*n* = 2,381) did not. The mean score of the PB scale was *M* = 1.59 (*SD* = 1.21), the mean score of the TB scale was *M* = 2.64 (*SD* = 1.36). Respondents reporting lifetime suicidal ideation scored significantly higher on all items of the INQ than participants who did not report suicidal ideation. Mean values on each item for both subgroups and results of the conducted t-tests are reported in Table 1. On average, on the PB scale persons with suicidal ideation showed values of *M* = 2.54 (*SD* = 1.70) while participants without suicidal ideation scored *M* = 1.18 (*SD* = 0.53). On the TB scale persons with suicidal ideation averagely showed values of *M* = 3.49 (*SD* = 1.46) while participants without suicidal ideation scored *M* = 2.27 (*SD* = 1.14).

**Table 1:** Mean values, standard deviations, t-tests, and effect sizes for the subsamples with and without suicidal ideation.

Item	Total sample (*N* = 3.404)		Subsample with suicidal ideation (*n* = 1.023)		Subsample without suicidal ideation (*n* = 2.381)		t-tests and effect sizes			
	*M*	*SD*	*M*	*SD*	*M*	*SD*	*t*	*df*	*p*	*d*
**Perceived Burdensomeness subscale**										
These days, the people in my life would be better off if I were gone. (1)	1.60	1.34	2.56	1.92	1.19	0.65	-22.28	1.125.15	< .001	1.18
These days, the people in my life would be happier without me. (2)	1.56	1.28	2.43	1.86	1.19	0.63	-20.83	1.125.05	< .001	1.15
These days, I think I am a burden on society. (3)	1.77	1.59	3.05	2.21	1.22	0.70	-26.00	1.111.02	< .001	1.34
These days, I think my death would be a relief to the people in my life. (4)	1.46	1.20	2.24	1.82	1.13	0.52	-19.14	1.095.10	< .001	1.09
These days, I think the people in my life wish they could be rid of me. (5)	1.44	1.13	2.11	1.69	1.16	0.59	-17.45	1.129.28	< .001	1.05
These days, I think I make things worse for the people in my life. (6)	1.70	1.50	2.88	2.12	1.20	0.64	-24.96	1.103.39	< .001	1.28
**Mean value**	1.59	1.21	2.54	1.70	1.18	0.53	-25.15	1.108.03	< .001	1.03
**Sum**	9.54	7.24	15.26	10.21	7.07	3.18	-25.15	1.108.03	< .001	6.19
Item	Total sample (*N* = 3.404)		Subsample with suicidal ideation (*n* = 1.023)		Subsample without suicidal ideation (*n* = 2.381)		t-tests and effect sizes			
	*M*	*SD*	*M*	*SD*	*M*	*SD*	*t*	*df*	*p*	*d*
**Thwarted Belongingness subscale**										
These days, other people care about me. (7)	2.53	1.74	3.06	1.92	2.30	1.60	-11.10	1.661.99	< .001	1.71
These days, I feel like I belong. (8)	2.91	1.96	3.96	2.02	2.45	1.74	-20.83	1.704.69	< .001	1.83
These days, I rarely interact with people who care about me. (9)	2.49	1.75	3.26	1.94	2.16	1.55	-16.06	1.608.63	< .001	1.67
These days, I am fortunate to have many caring and supportive friends. (10)	2.88	1.86	3.45	2.00	2.64	1.74	-11.31	1.715.34	< .001	1.82
These days, I feel disconnected from other people. (11)	2.15	1.69	3.42	2.07	1.61	1.11	-26.41	1.283.05	< .001	1.47
These days, I feel like an outsider in social gatherings. (12)	2.27	1.81	3.71	2.15	1.65	1.19	-28.89	1.301.92	< .001	1.54
These days, I feel that there are people I can turn to in times of need. (13)	2.67	1.90	3.07	1.93	2.50	1.86	-8.04	1.876.20	< .001	1.88
These days, I am close to other people. (14)	2.81	1.86	3.68	1.97	2.44	1.68	-17.55	1.689.83	< .001	1.77
These days, I have at least one satisfying interaction every day. (15)	3.02	1.93	3.84	2.09	2.67	1.75	-15.68	1.664.38	< .001	1.86
**Mean value**	2.64	1.36	3.49	1.46	2.27	1.14	-23.96	1.583.26	< .001	1.24
**Sum**	23.73	12.28	31.45	13.11	20.41	10.26	-26.38	3.402.00	< .001	11.19

### First network analysis

**Fig 1:**
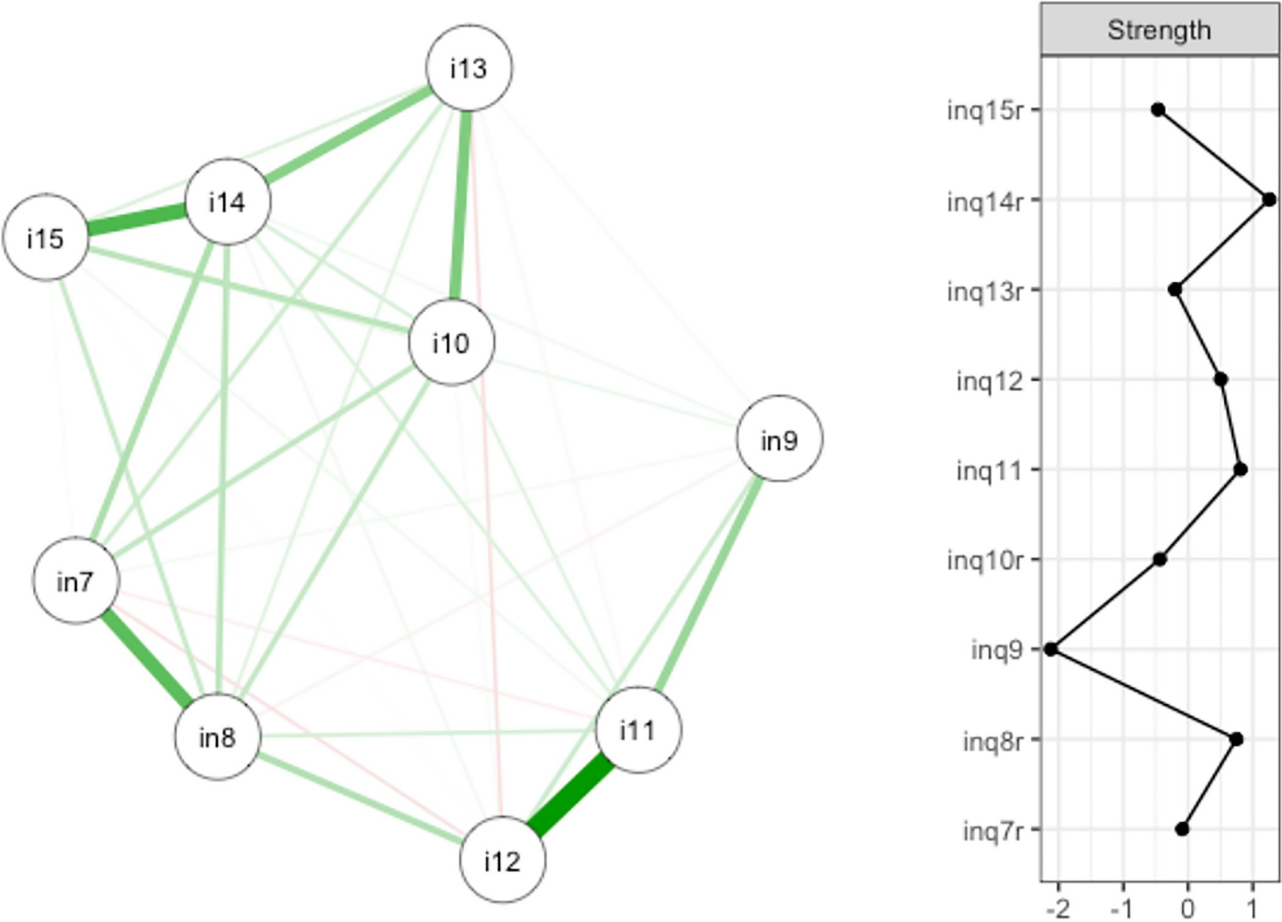
Network structure and strength of the TB scale in the INQ. The nodes represent the single items of the scale. Green connections show positive correlations, red connections show negative correlations. The thicker the edges are pictured, the stronger are the associations between two items. in7: These days, other people care about me. in8: These days, I feel like I belong. in9: These days, I rarely interact with people who care about me. i10: These days, I am fortunate to have many caring and supportive friends. i11: These days, I feel disconnected from other people. i12: These days, I often feel like an outsider in social gatherings. i13: These days, I feel that there are people I can turn to in times of need. i14: These days, I am close to other people. i15: These days, I have at least one satisfying interaction every day.

The visualization of the first network and its Strength is shown in Fig 1. The basic matrix consisted of the items of the TB scale.

The items with the largest Strength-indices were items 14 (*Z*_*S*_(14) = 1.26), 11 (*Z*_*S*_(11) = 0.81) and 8 (*Z*_*S*_(8) = 0.75). Item 10, which, according to the results of the considered factor analyses, should be one of the most central items, showed a negative Strength of *Z*_*S*_(10) = -0.44.

### Second network analysis

**Fig 2:**
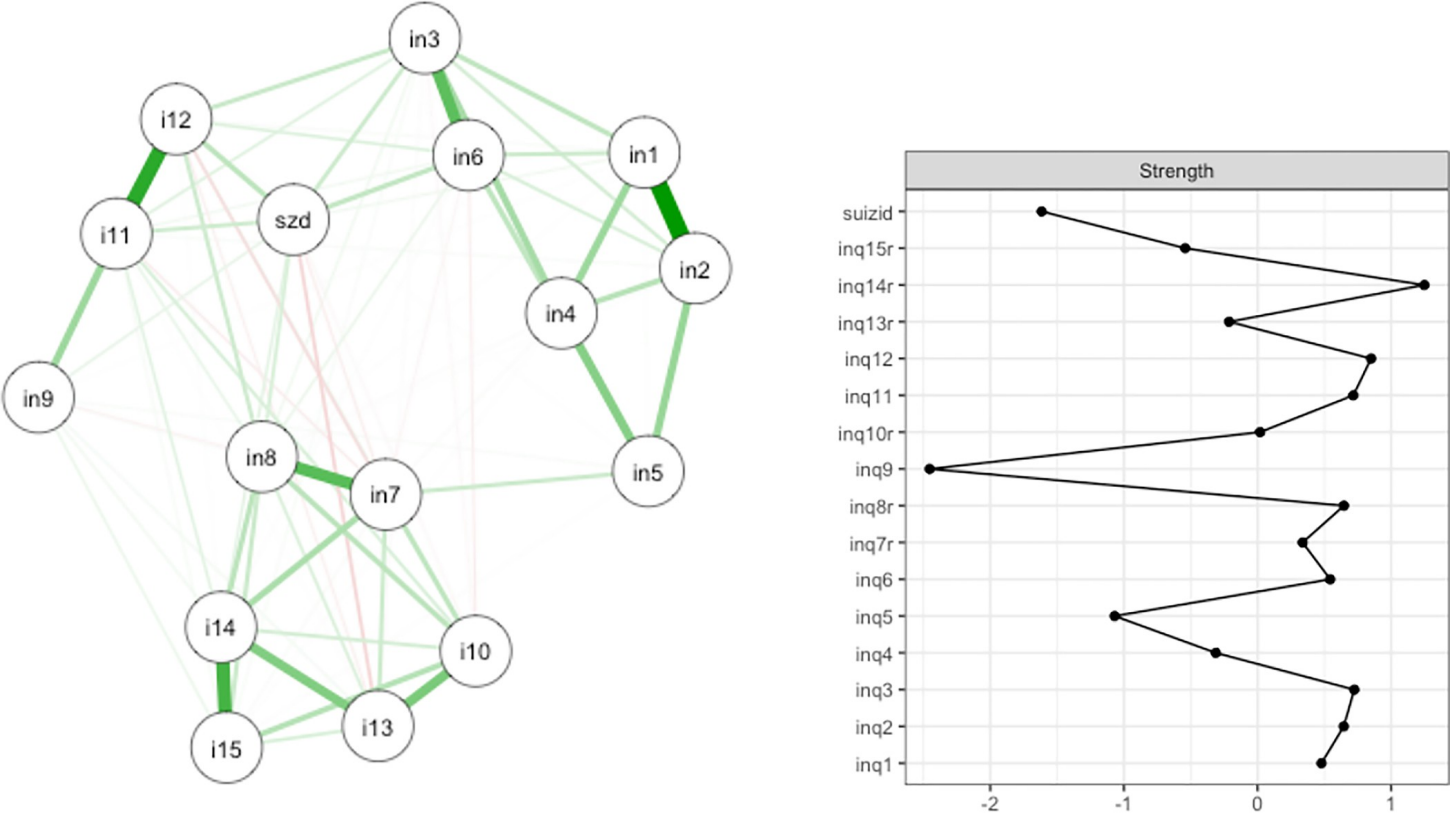
Network structure and strength of the total INQ and the suicide variable. The nodes represent the single items of the scale. Green connections show positive correlations, red connections show negative correlations. The thicker the edges are pictured the stronger are the associations between two items. in1: These days, the people in my life would be better off if I were gone. in2: These days, the people in my life would be happier without me. in3: These days, I think I am a burden on society. in4: These days, I think my death would be a relief to the people in my life. in5: These days, I think the people in my life wish they could be rid of me. in6: These days, I think I make things worse for the people in my life. in7: These days, other people care about me. in8: These days, I feel like I belong. in9: These days, I rarely interact with people who care about me. i10: These days, I am fortunate to have many caring and supportive friends. i11: These days, I feel disconnected from other people. i12: These days, I often feel like an outsider in social gatherings. i13: These days, I feel that there are people I can turn to in times of need. i14: These days, I am close to other people. i15: These days, I have at least one satisfying interaction every day. szd: suicide variable.

The basic matrix with the partial correlations contained several items of the INQ-15 as well as the variable representing suicidal ideation. The visualization of the network analysis is shown in Fig 2.

The strongest positive associations can be seen between items 1 and 2, 3 and 6, 7 and 8, 11 and 12, and 14 and 15, whereas weaker negative associations were found between item 13 and suicidal ideation, between items 7 and 12, and between items 7 and 11. The suicide variable showed the strongest positive associations with items 3, 6, 11, and 12. There were negative associations between the suicide variable and items 7, 10, and 13. Overall, there were stronger associations between the items within each scale than between the items of the two separate scales. The items with the largest Strength indices were item 14 (*Z*_*S*_(14) = 1.25), item 12 (*Z*_*S*_(12) = 0.85), item 3 (*Z*_*S*_(3) = 0.72), and item 11 (*Z*_*S*_(11) = 0.72). Item 9 (*Z*_*S*_(9) = -2.45) and the dichotomous suicide variable (*Z*_*S*_(11) = 0.72) have the lowest Strength indices compared to the average.

## Discussion

The aim of this study was to map the correlations of the items of the TB scale in the INQ by means of a network analysis and to present the items with the highest centrality. In addition, in a second network, the interrelationships of all items in the INQ with each other and with suicidal ideation were to be modeled in order to represent both the most central items and the items with the greatest connections to suicidal ideation.

As TB is a unidimensional scale according to the factor analyses, we could find high positive correlations between various items of the scale. Nevertheless, there were also several negative correlations, consisting of pairs with one negatively and one positively formulated item each. Since all factor analyses confirmed one superordinate factor for the TB scale, we additionally compared our results to a study by Upegui-Arango et al. [20] validating the psychometric properties of the INQ-15 with Item Response Theory methods. Their results are consistent with our findings as in their study, too, the authors had to exclude the three negatively formulated items 9, 11, and 12 from further analyses because of the scale’s lack of unidimensionality. So, our study as well as the other study excluded the same items. Consequently, there could be a method effect as a result of the wording of the items. These directional effects potentially appear in questionnaires depending on the wording of the items [48]. Thus, there is a possibility that such an effect was revealed in the network analysis and the analysis of Upegui-Arango et al. [20]. This suggests that it might be possible that different approaches to test the psychometric properties of the INQ-15, such as factor analyses, potentially lead to different results. The starting point for a factor analysis is a correlation matrix that contains all correlations between the observed manifest variables [49]. This can lead to spurious correlations, i.e. the apparently causal connection between two variables is originally caused by a third variable [50]. For a network analysis, on the other hand, a matrix consisting of partial correlations usually serves as the basis [46]. Partial correlations can reveal and eliminate spurious correlations between variables [50].

Moreover, there may also be a content-related reason for the items of the TB scale showing negative correlations among each other in the network. The three items 9 ("These days, I rarely interact with people who care about me."), 11 ("These days, I feel disconnected from other people.") and 12 ("These days, I feel like an outsider in social gatherings.") may represent a different part of the construct TB than the other items, as their content could be more about a feeling of *being cut off from* other people. In contrast, the remaining items 7, 8, 10, 13, 14, and 15 seem to represent the *need to belong* [8]. In particular, item 8 ("These days, I feel like I belong."), which was also one of the most central items in the network, seems to play a decisive role here. In the first network these items similarly correlate positively with each other, whereas the three items 9, 11 and 12 form a separate cluster. It is therefore possible that they build—at least—two different clusters that further subdivide the construct TB in terms of content. The ITPS equally subdivides the construct into two dimensions—*Loneliness* and *Absence of Reciprocal Care* [7]. In this case, loneliness is similar to the feeling of being cut off from other people (items 9, 11, 12) and the need to belong is similar to the absence of reciprocal care (items 7, 8, 10, 13, 14, 15). In terms of content, the Absence of Reciprocal Care dimension seems very similar to the PB scale: someone who does not receive any attention from their social environment may sooner feel like a burden. Conversely, a person who feels like a burden will seek less help and consequently receive less attention.

The first network demonstrated the items 8 and 14 to be the most central ones, for the most part confirming our assumptions based on the network analysis. However, in the second network we found high correlations between the suicide variable and items 11 and 12. Item 11 was central in both networks. Item 12 is an item that was not central in the first network with the TB scale but showed very high centrality in the second network. Item 14, which was central in both networks, showed only small positive correlations with the suicide variable in the first network. Item 10, which was expected to be central based on results of factor analyses in prior studies, even showed a negative correlation with the suicide variable. Regarding the PB scale, the suicide variable showed high correlations with items 3 and 6: According to the Strength, item 3 was one of the most central items in the network, whereas item 6 was less central. Thus, centrality in the network does not necessarily imply a substantial correlation with suicidal ideation. In fact, less central items in the TB network may also highly correlate with suicidal ideation and, conversely, very central items in the TB network may correlate not at all or even negatively with suicidal ideation.

### Implications for practice

Overall, the associations between TB and PB with suicidal ideation have been confirmed. Nevertheless, the network analyses demonstrated that the items with the highest centrality within the network restricted of items indicating the respective construct (e.g., TB) are not necessarily those that show the highest correlations with suicidal ideation. We also found that not all items of the INQ-15 correlated equally with suicidal ideation. A recent study by Brown et al. [51] showed similar results. In a network analysis with the INQ-15, the authors found that items 3 and 8 were most influential for suicidal ideation. Considering that the sum scores of the subscales TB and PB are often used to predict suicidal ideation, there is one specific problem: A high score on one of the subscales may not necessarily imply intense suicidal ideation when a person only scores high on the items that only weakly correlate with suicidal ideation. In contrast, a low score on one of the subscales may conceal intense suicidal ideation when a person only scores high on the few items that show high connections with suicidal ideation but yields a low total score. Brown et al. [51] thus also suggest that TB and PB should be assessed by using only a limited number of items of the INQ. Therefore, it might not be necessary to present the entire INQ to assess concrete suicide risk. Rather, those items of the two scales assessing PB and TB with the highest correlations with suicidal ideation could be used. These items are item 3 ("These days, I think I am a burden to society.") and item 6 ("These days, I think I make things worse for the people in my life.") from the PB scale, and item 11 ("These days, I feel disconnected from other people.") and item 12 ("These days, I feel like an outsider in social gatherings.") from the TB scale. This results in a shortened and economical version of the INQ consisting of two items from each scale. Consequently, this shortened version of the INQ does not contain any items from the Absence of Reciprocal Care dimension. However, since this dimension is similar to the PB scale in terms of content as discussed above, it is nevertheless taken up and covered in the suggested shortened version.

An abbreviated scale has potential advantages of further reducing fear of stigmatization, hospitalization, being medicated, or concerns that others may be informed about the person’s suicidality [23]. By asking only four of the 15 total questions, this could further reduce face validity. Thus, eventually, the willingness to answer the questions honestly might also increase. Consequently, suicidal ideation can be inferred more accurately and quickly from answering the four questions than is the case when answering the entire INQ. Thus, the shortened version of the INQ could initially serve as a screening instrument: if patients score high in the survey, further diagnostics, especially in the form of personal interviews, can be carried out to explicitly assess the individual risk of suicide. However, the INQ cannot be used as a diagnostic tool for any diagnoses according to the ICD-10 [52] or DSM-IV [53]. Finally, the complete scale also consists of items that are unrelated or even negatively related to suicidal ideation. According to the network analyses, these items provide little or no added information about a patient’s suicidality.

A short version of the INQ has one significant benefit: it can be easily integrated in the measurement of suicidal ideation within the framework of Ecological Momentary Assessment (EMA) studies. As suicidality is a fluctuating phenomenon [e.g., 54], EMA is a useful approach to examine short-term predictors of suicidality in real-time for specific time periods [55]. Participants use mobile devices to frequently complete assessments at fixed or random times of the day, so it can be useful to monitor individuals with suicidal ideation [56]. The intensity of suicidal ideation can fluctuate drastically within short periods of time [57]. Thus, a short version of the INQ with only four items addressing the two predictors of suicidal ideation TB and PB could bear useful information for both mental health clinicians and scientists. Based on this information, it is then possible to work with the patients on their individual experience of TB or PB, either interventionally or preventively in psychotherapy. For example, studies [58] show that both constructs are related to the need to belong, offering different starting points for psychotherapeutic interventions.

### Strengths and weaknesses of the study

The network approach to psychopathology assumes that symptoms of a disorder interact with each other and do not represent individual, independent components of a superordinate diagnosis. Thus, the application of network analysis to research on suicidality can lead to a better understanding of suicidal behavior as the network perspective can validate complex explanatory models such as the IPTS [27]. Hence, it was possible to map the relationships of the different items of TB and PB with each other, as well as the correlations of the individual items with suicidal ideation. We demonstrated that not all items of the scales correlate equally with each other and with suicidal ideation and thus vary regarding their predictive validity. To conduct the network analyses, the INQ was used as a basis. The INQ is a widely tested, reliable and valid instrument for measuring TB and PB [34]. The German version used here was established as an empirically derived improvement of the original version INQ-25 [11] with consistently stable psychometric properties across studies [21,33]. Finally, the network analysis was based on a large sample (*N* = 3,404) that included both individuals with (*n* = 1,023) and without (*n* = 2,381) suicidal ideation.

Despite the advantages of generating the sample from different source data (general population, clinical practice), this approach also contains disadvantages. First, the study hypotheses and the procedure have not been pre-registered. Aside from that, measuring suicidality with different instruments did not allow a uniform representation of the respondents’ suicidality. Thus, it was also not possible to verify whether the persons were acutely suicidal or not. The correlation between current suicidality and the INQ could yet provide more information about the most significant items for assessing suicidality. A more detailed and uniform assessment of suicidality is therefore warranted.

## Conclusion

The main goals of this study were to map the correlations of the items the INQ by means of two network analyses and to present the items with the highest centrality. Moreover, a network mapping the interrelationships of the items in the INQ with suicidal ideation was modeled to represent the items with the greatest connections to suicidal ideation.

It was possible to expose those items of the INQ-15 with the highest links to suicidal ideation. It turned out that the items that show the highest centrality in the networks were not necessarily those items with the strongest connections to suicidal ideation. Moreover, the items that showed the highest factor loadings according to the factor analyses in previous studies were not necessarily the items that correlated most highly with suicidal ideation. As the INQ is primarily used to predict suicidal ideation in clinical research and practice, a proposal for a shortened version of the INQ with the four items that showed the greatest associations with suicidal ideation in the network analyses was made. However, further research on different populations with and without suicidal ideation, acute as well as non-acute, is needed to validate the results. Additional network analyses with the full item set of the INQ are also recommended to identify all items with high associations to suicidal ideation.

## Supporting information

S1 Dataset (XLSX)
